# Drug-Induced Liver Injury by Glatiramer Acetate Used for Treatment of Multiple Sclerosis: A Case Report

**DOI:** 10.1177/2324709613517493

**Published:** 2013-12-17

**Authors:** Attila Onmez, Ahmet Tarik Eminler, Hasan Ergenç, Meltem Baykara, Ihsan Uslan, Ali Tamer

**Affiliations:** 1 Sakarya University Training and Research Hospital, Department of Internal Medicine, Sakarya; 2 Sakarya University Training and Research Hospital, Department of Gastroenterology, Sakarya

**Keywords:** glatiramer acetate, multiple sclerosis, toxic hepatitis

## Abstract

Glatiramer acetate (GA, Copaxone) is an approved drug for the treatment of relapsing–remitting multiple sclerosis. Most common side effects observed with GA are local injection site reactions, which can include pain, swelling, or redness. However, systemic adverse event such as hepatotoxicity related to GA is rarely seen. In this report, we present a case of GA-induced toxic hepatitis associated with cholestatic and hepatocellular damage.

## Introduction

Glatiramer acetate (GA) is a synthetic polypeptide composed of 4 amino acids (l-tyrosine, l-glutamic acid, l-alanine, and l-lysine) resembling myelin basic protein.^1^ GA was approved by the US Food and Drug Administration as a treatment for patients with active relapsing–remitting multiple sclerosis (MS) in 1997. However, its mechanism of action still remains unclear. Several mechanisms have been reported in the literature. Administration of GA shifts the population of T cells from pro-inflammatory Th1 cells to regulatory Th2 cells that cross the blood–brain barrier and suppresses the inflammatory response.^2^ GA has a high safety profile, and it is tolerated well by patients.^3^ Local injection site reactions are observed more often than systemic adverse events in the treatment process.^4^ GA-induced toxic hepatitis is extremely rare. In this article, we present a case of GA-induced severe hepatotoxicity.

## Case Report

A 36-year-old woman was referred to our clinic from a regional hospital where she had been admitted 11 days earlier with symptoms of fatigue, nausea, dark-colored urine, and jaundice. Her medical history revealed that she had been followed-up for MS for 4 months and was prescribed subcutaneous 20 mg/day GA treatment for 1 month. She was given steroids for 3 months after the diagnosis. However, she had never been treated with interferon. She did not have any disease other than MS. She does not drink alcohol or smoke. The patient denied the use of illicit drugs or herbal remedies. On physical examination, the patient had jaundice, scleral icterus, and mild discomfort on liver palpation; hepatosplenomegaly, ascites, or flapping tremor was not detected.

Laboratory examination revealed the following results: aspartate aminotransferase, 1834 IU/L (reference = 0-32); alanine aminotransferase, 1475 IU/L (0-32); γ-glutamyl transferase, 46 IU/L (5-36); alkaline phosphatase, 231 IU/L (35-105); total bilirubin, 24.4 mg/dL (0-1.2); direct bilirubin, 19.38 mg/dL (0-0.3); prothrombin time, 14.9 seconds (9.4-12.5); and fasting glucose, total protein, albumin, globulin, amylase, and lactate dehydrogenase levels were within normal limits. Complete blood count was normal, and urine analysis revealed 3 positive urobilinogens. The liver enzyme levels when she started GA treatment were within normal limits. Serologic tests for hepatitis A, B, and C; cytomegalovirus; and Epstein–Barr virus were negative. Autoimmune hepatitis markers, antinuclear antibody, anti-smooth muscle antibodies, antimitochondrial antibody, and liver kidney microsomal antibody results were negative. Serum iron, ferritin, copper, ceruloplasmin, α1-antitrypsin levels, and thyroid function test were normal. Abdominal ultrasonography of the liver and biliary tract was also normal. There was no ascites or splenomegaly observed.

**Figure 1.**
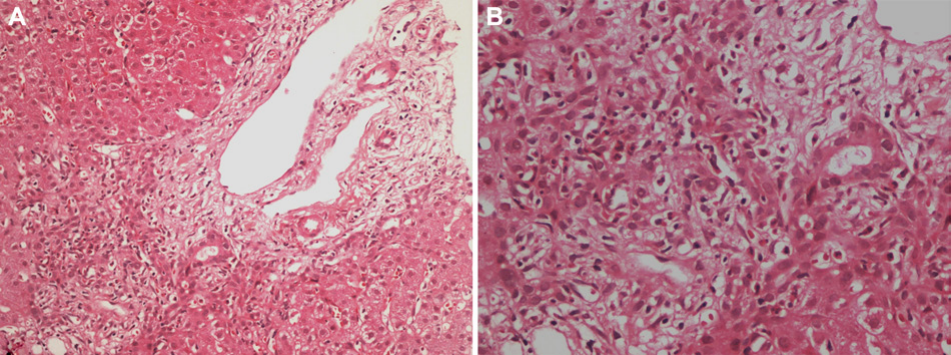
(A) Fibrous portal expansion, bile duct proliferation, and a mixed inflammatory infiltrate predominantly consisting of polymorphonuclear leukocytes, partially disrupting the limiting plate are seen (hematoxylin–eosin; magnification 200×). (B) Mixed inflammatory reaction in portal tract and parenchyma, predominantly consisting of polymorphonuclear leukocytes; number of eosinophils, few lymphocytes, and plasmocytes are also seen in this field (hematoxylin–eosin; magnification 400×).

**Figure 2.**
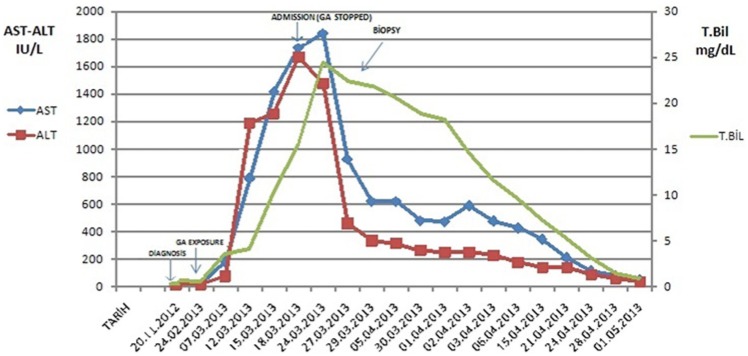
Serum transaminase levels during admission and follow-up. Abbreviations: AST, aspartate aminotransferase; ALT, alanine aminotransferase; T.Bil, total bilirubin.

On the first day of admission, GA was discontinued at our clinic. The previous clinic investigated other causes of jaundice and GA had not ceased. We performed routine monitoring of vital signs. Hydration was achieved with parenteral fluids. Three days after the discontinuation of GA, liver enzyme levels showed a tendency to decrease. Fifteen days after the clinical evolution, a liver biopsy was performed, which revealed fibrous expansion of the some portal area and bile duct proliferation. The limiting plate was disrupted by polymorphonuclear-rich mixed-type inflammatory cell reaction (Figure 1). The patient’s liver function tests returned to normal levels within a period of 36 days (Figure 2).

The score of the Roussel Uclaf Causality Assessment Method for drug-induced liver injuries also revealed a probable association.

## Discussion

Drug-induced liver injury is a common liver disease that generally occurs between 5 and 90 days after drug ingestion. The clinical picture of the disease is variable, ranging from transient mild elevation of liver enzymes to fulminant liver failure leading to death.^5^

One of the major mechanisms that can lead to toxic effects on the liver is the intrinsic mechanism. This is predictable, dose-dependent, and has characteristic emergence with the intake of certain drug overdose. The second one is defined as idiosyncratic, which is the most common form of hepatotoxicity and is characterized with unpredictable reactions. Variations according to the individual differences in drug metabolism as well as genetic and environmental factors are also important. This type of toxic reaction is not dose-dependent and cannot be displayed by experimental studies.^6^

Glatiramer acetate is generally well tolerated by the patients. The most common side effects observed with GA are local injection site reactions, which can include pain, swelling, or redness. Systemic adverse event related to GA is rarely seen, but postinjection transient flushing, chest tightness, palpitations, and dyspnea can be observed.^7^

In the literature, some studies claimed that GA does not cause liver enzyme abnormalities.^8^ However, there are 2 previous reports of GA-induced toxic hepatitis. The first case was of a 52-year-old woman who presented with hepatocellular type toxic hepatitis, and thus cholestatic enzyme levels were normal. The liver biopsy was compatible with drug-induced hepatitis. The aminotransferase levels returned to normal after drug discontinuation. In this patient, initially identified antinuclear and anti-smooth muscle antibodies disappeared or were tested at low titers when GA was discontinued.^9^ The second report involved a 31-year-old female who was admitted with anorexia, lethargy, and jaundice 5 weeks after commencing GA treatment. She had abnormal liver tests. The liver biopsy consistent with drug toxicity, and liver tests returned to normal within 2 months after the discontinuation of GA.^10^ In our case, other causes of liver injury were excluded, and abnormal liver enzyme levels returned to normal levels within 2 months after the discontinuation of the medication. We performed liver biopsy to confirm the diagnosis, and the biopsy was compatible with drug-induced hepatitis.

Glatiramer acetate was suggested to induce autoimmune disease. It is believed that GA induces T helper type 2 cells that cross-react with myelin basic protein and may enhance the production of autoantibodies. Therefore, it can be speculated that GA induces autoimmune side effects.^9^ In 2007, a case of triggered autoimmune hepatitis with GA was reported.^11^ But, like our case, the patients in the 2 previous case reports had negative autoimmune markers, and the biopsies showed no features of autoimmune disease. So we can define this effect as idiosyncratic.

The cases reported earlier and our patient were women, which is an interesting point worthy of further research.

In conclusion, physicians should keep in mind the possibility that GA may induce severe hepatotoxicity. This adverse event is extremely rare. But other immunmodulators such as interferons, which are prescribed for MS patients, may likely cause these toxic effects. So we can suggest acquiring the baseline liver enzyme level prior to GA treatment. Also, we recommend close monitoring of liver enzymes in patients with MS who use immunmodulators.
